# Immunomodulatory Activity of the Marine Sponge, *Haliclona* (*Soestella*) sp. (Haplosclerida: Chalinidae), from Sri Lanka in Wistar Albino Rats: Immunosuppression and Th1-Skewed Cytokine Response

**DOI:** 10.1155/2020/7281295

**Published:** 2020-11-12

**Authors:** Varuni Gunathilake, Marco Bertolino, Giorgio Bavestrello, Preethi Udagama

**Affiliations:** ^1^Department of Zoology and Environment Sciences, Faculty of Science, University of Colombo, Colombo 3, Sri Lanka; ^2^Dipartimento di Scienze della Terra, Dell'Ambiente e della Vita, Università degli Studi di Genova, Corso Europa 26, 16132 Genova, Italy

## Abstract

Natural secondary metabolites of sponges of the genus *Haliclona* are associated with an array of biological activity with therapeutic usage. We investigated the immunopharmacological properties of a presumably novel marine sponge species from Sri Lanka, *Haliclona* (*Soestella*) sp. Sponge material was collected from southern Sri Lanka by scuba diving. Sponge identification was based on spicule and skeleton morphology using light microscopy. Selected *in vivo* and *ex vivo* tests investigated nonfunctional and functional immunomodulatory activity of the *Haliclona* (*Soestella*) sp. crude extract (HSCE) in the Wistar rat model. Compared to the controls, rats orally gavaged daily for 14 consecutive days with 15 mg/kg dose of the HSCE manifested a significant reduction of immune cell counts of total WBCs (by 17%; *p* < 0.01), lymphocytes (38%), platelets (52%), splenocytes (20%), and bone marrow cells (BMC; 60%) (*p* < 0.001), with a concurrent increase in the neutrophil : lymphocyte ratio (*p* < 0.05); RBC counts abated by 53% (*p* < 0.001). A significant reduction of the splenosomatic index was evident with the 10 and 15 mg/kg doses (*p* < 0.001). Rat plasma TNF-*α* cytokine level was augmented by tenfold (*p* < 0.001), IL-6 level by twofold (*p* < 0.01) with the 15 mg/kg HSCE treatment, while IL-10 was detectable in rat plasma only with this treatment; the corresponding Th_1_ : Th_2_ cytokine ratio (TNF-*α* : IL-10) was indicative of an unequivocal Th1-skewed cytokine response (*p* < 0.01). *Ex vivo* bone marrow cell and splenocyte proliferation were significantly and dose dependently impaired by HSCE (IC_50_ 0.719 and 0.931 *μ*g/mL, respectively; *p* < 0.05). Subacute toxicity testing established that HSCE was devoid of general toxic, hepatotoxic, and nephrotoxic effects. In conclusion, HSCE was orally active, nontoxic, and effectively suppressed nonfunctional and functional immunological parameters of Wistar rats, suggestive of the potential use of the HSCE as an immunosuppressant drug lead.

## 1. Introduction

Marine pharmacognosy has gained much attention in the recent years due to the vast biological and chemical diversity of marine organisms [1, 2]. Of all marine invertebrates, marine sponges are particularly amongst the abundant reserves of novel natural products with distinct biological activity of pharmaceutical importance [3]. Several drug discovery and development programs are currently focused on the search for bioactive compounds from marine sponges; these organisms provide novel drug leads with antibacterial, antiviral, antifungal, antimalarial, antitumor, immunosuppressive, and cardiovascular activity and for many other diseases including cancers [4]. This has resulted in the inclusion of a considerable number of sponge-derived drugs, in the global marine pharmaceutical pipeline [5].

Immune system dysfunction leads to the development of hypersensitivity reactions, autoimmune diseases, and chronic inflammatory diseases. Immunotherapies induce, enhance, or suppress an immune response to ameliorate a pathological condition [6]. Drugs and nutrients can function as immunostimulants, to enhance the activity of both innate and adaptive immune components. Immunoadjuvants for example incline innate and adaptive immunity through vaccines for conditions such as cancers or infection. Immunosuppression therapies can be used to prevent graft rejection and treat autoimmune conditions and allergies. Recent estimates suggest that 7.6–9.4% of the world's population is affected by immune-mediated diseases such as inflammatory bowel disease (IBD), type 1 diabetes mellitus (TIDM), and rheumatoid arthritis (RA) [6]. Autoimmune diseases are among the ten leading causes of death for women [7], rendering the combat of immune-related diseases, a global challenge. Therefore, a critical need exists for such novel drugs with the potential to restore immune homeostasis of a dysfunctional immune system; hence, bioprospecting for such drug leads is crucial.

Marine sponges are a highly diversified group, and their secondary metabolites are biologically active and chemically unique. To date, a number of potential immunomodulators, which can be used as biochemical or structural chemophores to develop therapeutics, have been isolated from sponges. New biomolecules discovered from marine sponges have strong immunosuppressive activity [7]. For example, polyoxygenated sterols derived from *Dysidea* sp., have been shown to have strong selective immunosuppressive capability, blocking the interaction between IL-8 and its receptor [8]. Similarly, pateamine A from *Mycale* sp. and discodermolide from *Discodermia dissolute* have shown inhibitory properties on the production of IL-2 in T and B lymphocytes and unique immunosuppressive and cytotoxic properties, respectively [6]. Immunosuppressive properties of discodermolide at low concentrations were further proven by both *in vivo* and *in vitro* measures [9]. Simplexides, a new class of glycolipids, are strong immunosuppressors *in vitro* and have been isolated from the marine sponge *Plakortis simplex* [10].

A comprehensive list of previous reports on immunosuppressive activity of sponges is provided in recent reviews [4, 6, 11]. Accordingly, in the late 1980s, two immunosuppressive compounds were isolated from a deep water marine sponge, *Agelas flabellrformis*, 4a-merhyl-5a-cholest-8-en-3~-ol and 4,5-dibromo-2-pyrrolic acid with important immunosuppressive activity. Both compounds were highly active in suppression of the response of murine splenocytes with little to no demonstrable cytotoxicity at low doses. Three polyoxygenated sterols from a *Dysidea* sp. from Australia, which are selective immunosuppressive compounds that inhibit the binding of IL-8, were reported. The simplexides from the Caribbean sponge *Plakortis simplex* are a group of immunosuppressive glycolipids that inhibit proliferation of activated T cells. Pateamine A, from a *Mycale* sp., inhibits the production of IL-2 and thereby the activation of resting T cells and B cells to a lesser extent. Contignasterol from *Petrosia contignata* inhibits allergen-induced histamine release from rat mast cells and from guinea-pig lung tissue *in vitro*. Discodermolide, a unique immunosuppressive and cytotoxic agent, isolated from the deep water sponge, *Discodermia dissolute*, reported a plethora of immunosuppressive activity.

Sponges of the family Haliclonidae are rich sources of nitrogen containing metabolites with various biological activities [12, 13]. The genus *Haliclona* is well known for producing a variety of secondary metabolites, most commonly bioactive alkaloids [12]. Bis-1-oxaquinolizidine alkaloids and furano sesquiterpene herbacin were reported from various *Haliclona* species [12]. They also produce metabolites with diverse structures including polycyclic amines, sesquiterpenoids, quinols, glycosphingolipids, resorcinol, and tetrahydropyranol [12]. The high chemical and structural diversity of secondary metabolites produced by this genus result in interesting biological activity such as cytotoxic, antifungal, antibacterial, antiviral, antimalarial, anti-inflammatory, neuritogenic, and hemolytic activities [12].

Sri Lanka, as one of the world's richest biological hotspots, abundantly harbours a diversified marine sponge fauna, in large part, until now unexplored with respect to both their biodiversity and zoochemical constituents. A few bioactivities of Sri Lankan marine sponge extracts were reported such as human sperm immobilization activity [14], yet a considerable research gap exists in this field. The present study was undertaken to bridge this knowledge gap. The crude extract of the sponge, *Haliclona* (*Soestella*) sp. (HSCE) was investigated for its immunomodulatory potential by means of nonfunctional (immune cell counts and cytokine levels) and functional (cell proliferation) immunological parameters in the Wistar albino rat model. Further, liver and kidney function parameters and stress parameters were examined to investigate the safety of the oral administration of HSCE.

## 2. Materials and Methods

### 2.1. Sponge Sampling

With the approval of the Department of Wildlife, Sri Lanka (WL/3/2/1/6), *Haliclona* (*Soestella*) sp. was collected from Unawatuna, Galle, Sri Lanka (6°01′N 80°15′E) by scuba diving at a depth of 9-19 m of shallow waters, facilitated by the National Aquatic Resources Research and Development Agency (NARA), Sri Lanka. Samples were packed in ice during transportation to the laboratory and stored at -20°C until extraction.

### 2.2. Identification of *Haliclona* (*Soestella*) sp.

Sponge sample was authenticated at the Department of Science, Environment and Life (DISTAV), University of Genoa, Italy, while a type specimen was deposited in the Department of Zoology and Environment Sciences, University of Colombo, Sri Lanka (Code -2G). Sponge identification was carried out using morphology, spicule, and skeleton analyses. For the preparation of spicules, a small fragment of the preserved sponge sample was dissolved in 65% nitric acid, both in test tubes and directly on glass microscope slides. Next, these were rinsed with water, dehydrated in 90% ethanol, and finally mounted in Eukitt resin (Fluka, Italy). Tangential and transverse sections, cut manually with a blade, from partly dehydrated specimens, were mounted in Eukitt resin to study the skeletal architecture by light microscopy. Sponge species were identified according to the guidelines provided by Hooper and Soest and classified under the updated nomenclature provided in the World Porifera Database [15].

### 2.3. Animal Model

Healthy male adult Wistar albino rats weighing 180-200 g (*n* = 35), purchased from the Medical Research Institute, Colombo, were used with ethical approval from the Faculty of Medical Sciences, University of Sri Jayewardenepura (No: 640/12). Animals were housed in polypropylene cages under standard animal house conditions at 25 ± 1°C room temperature and with pelleted food and water *ad libitum* throughout the experiment. They were treated and used in experiments adhering to ethical practices outlined in the OECD guidelines [16].

### 2.4. Preparations of the *Haliclona (Soestella)* sp. Sponge Crude Extract (HSCE)

Sponge samples preserved at -20°C were rinsed with running tap water. Thereafter, diced sponge material (approximately 2 cm fragments) was extracted first in methanol for 24 hrs, followed by 24 hrs in dichloromethane and finally in methanol : dichloromethane (1 : 1 v/v) for another 24 hrs, filtered through Whatman No. 1 filter paper, and subjected to rota evaporation (R 200-USA) [17]. The HSCE in powder form was dissolved in 5% ethanol and orally gavaged to adult male Wistar rats (*n* = 6/group), once daily for 14 consecutive days at appropriate doses (5, 10, and 15 mg/kg of body weight). Rats in the control group (*n* = 6) were provided 5% ethanol as the vehicle. Concentrations of 10, 100, 500, 1000, and 2000 *μ*g/mL of the HSCE were used for *ex vivo* assays.

### 2.5. Effect of the HSCE on Nonfunctional Immunological Parameters

Nonfunctional immunological parameters were used to test for the alterations in the immune cell profiles [total white blood cell (WBC) and differential WBC counts; neutrophil : lymphocyte ratio; and platelet count], cellularity based on splenocyte and bone marrow cell (BMC) counts, splenosomatic index (spleen/body weight ratio), and plasma cytokine [interferon gamma (IFN-*γ*), tumour necrosis factor alpha (TNF-*α*), interleukin-6 (IL-6), and interleukin-10 (IL-10)] levels.

At the end of the fortnight treatment with the HSCE, animals were sacrificed by an overdose of diethyl ether; rat blood was collected into EDTA containing tubes by heart puncture [17]. Blood diluted in Turk's solution was used in a Neubauer hemocytometer to obtain total WBC counts [17]. Differential WBC counts were made using Giemsa stained thin blood smears observed under oil immersion microscopy (×100). Further, diluted anticoagulated blood in freshly prepared 1% ammonium oxalate was used to obtain platelet counts [17]. Spleen weight was recorded using an electronic balance (±0.0011 g, EB-3200H-A, Shimadzu Corporation, Japan) and the spleen weight : body weight ratio (SW : BW) was calculated. The total splenocyte and BMC counts of the left femur of rats were calculated using a Neubauer hemocytometer [17].

Rat plasma was prepared by centrifugation of freshly collected nonheparinized blood at 750 x *g* for 10 minutes [17]. Plasma cytokine concentrations were assayed using standard sandwich ELISA kits designed for rats, following the manufacturer's instructions, for IFN-*γ*, TNF-*α*, IL-6, and IL-10 (BD Opt EIA^TM^, BD Bioscience, USA). In brief, ELISA plates were coated with the relevant capture antibody (anti rat IFN-*γ*, TNF-*α*, IL-6, and IL-10). Subsequent to blocking the plates with assay diluent (10% fetal bovine serum in PBS) fresh rat plasma was added and incubated. After washing, the plates were interacted with the detection antibody (Biotinylated anti rat IFN-*γ* for IFN-*γ* analysis etc.), followed by the addition of enzyme conjugate [avidin-horseradish peroxidase (HRP)] and the chromogen, O-phenylaminedichloride (OPD). Optical density was measured at 490 nm, using an microplate reader (Model 680, BioRad, USA). The concentration of each cytokine was calculated from a standard curve constructed with cytokine standards provided in the kits.

### 2.6. *Ex Vivo* Proliferation of Rat BMCs and Splenocytes

Outcome of BMC and splenocyte proliferation tests are listed under the purview of functional immunological parameters. The MTT dye reduction assay investigated the *ex vivo* proliferation of BMCs and of splenocytes [18, 19]. Briefly, 20 *μ*L of various concentrations (10, 100, 500, 1000, and 2000 *μ*g/mL) of the HSCE and the control (5% ethanol) were added to 20 *μ*L of cell suspension (1 × 10^6^ cells/mL) and 40 *μ*L of complete RPMI medium, in a 96-well plate. Proliferation of BMCs/splenocytes in the absence of mitogens was investigated. The cells were incubated at 37°C in a humidified 5% CO_2_ atmosphere for 48 h. Subsequently, 20 *μ*L of MTT (5 mg/mL) in PBS and 40 *μ*L of RPMI were added. The culture medium was removed by aspiration and 100 *μ*L of 0.04 M hydrochloride acid (HCl) in isopropyl alcohol was added to lyse the cells. Finally, 100 *μ*L of distilled water was added to each well, and the absorbance was measured at 570 nm using a microplate reader (Model 680, Bio-Rad, USA). The percentage cell proliferation was calculated for each cell type using the following equation [18, 20]:

(1) $$\begin{array}{c} \text{Cell}\text{proliferation}\left(\%\right)=\frac{\left(\text{OD}\text{of}\text{treated}‐\text{OD}\text{of}\text{control}\right)}{\text{OD}\text{of}\text{control}}\times 100\%. \end{array}$$

### 2.7. *Ex Vivo* Cytokine Production by Rat BMC Primary Cell Cultures

BM cellular suspensions of Wistar rats were established as described above but were stimulated by adding 20 *μ*L of heat-inactivated yeast (*Saccharomyces cereviceae*) per well [21]. After 48 hours of incubation, the supernatants with the nonadherent cells were centrifuged at 1000 x *g* at 4°C for 10 min. The resultant supernatants were assayed by sandwich ELISA to determine levels of IFN-*γ*, TNF-*α*, IL-6, and IL-10 rat cytokines.

### 2.8. Evaluation of Subacute Toxicity of the HSCE

Eight rats were randomly divided into two groups (*n* = 4/group); one group was treated orally for 14 consecutive days with the highest dose (15 mg/kg) of the HSCE to determine its subchronic toxicity [16]. The control group received 5% ethanol. During this period, the animals were observed for overt signs of toxicity (salivation, diarrhea, tremor, ataxia, yellowing of fur, loss of fur, lethargy, sleepiness, postural abnormalities, or behavioral changes), stress (fur erection and exophthalmia), and aversive behavior (biting of paw, intense grooming behavior, scratching behavior, and licking of tail) [22]. Body weights of the animals were determined pre and post treatment to check for possible weight loss. Blood samples were collected, and hematotoxicity was investigated by enumerating total RBC, total WBC, WBC differential, and platelet counts. Serum was separated and used to test for hepatic parameters [alanine aminotransferase (ALT), aspartate aminotransferase (AST), serum protein level, and bilirubin] using standard kits (Randox, USA). Nephrotoxicity was evaluated by analyzing serum creatinine and urea levels (Randox, USA), while serum cortisol level was quantified to assess stress levels using a rat sandwich ELISA kit (Human, USA).

### 2.9. Statistical Analyses

Data were expressed as means ± SEM. Statistical comparisons were made using the SPSS-20 software (IBM, USA) with the 5% ethanol control, for immunomodulatory tests using Mann-Whitney *U* test. Data generated *ex vivo* was analyzed by analysis of variance (ANOVA), followed by Tukey's test. The level of significance was set at *p* < 0.05.

## 3. Results

### 3.1. Description of the Test Sponge Specimen

**Figure 1:**
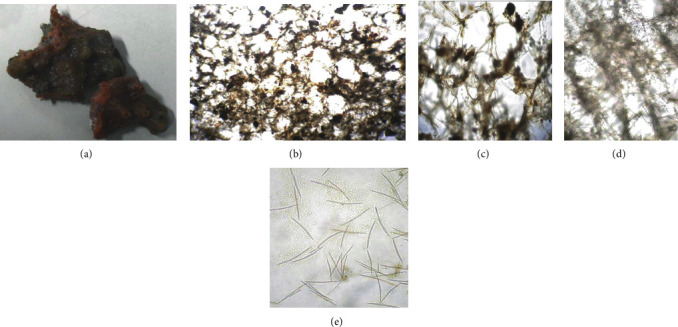
Photographs and micrographs of sponge morphological characters: (a) *Haliclona* (*Soestella*) sp. showing an irregular growth form; (b, c) cross sections of the ectosomal skeleton displaying multispicular polygonal meshes (400x); (d) cross section of the coanosomal skeleton, with irregular reticulation of primary lines ascending toward the surface, connected by unispicular secondary lines (400x); (e) oxeas of various sizes (1000x).

The sponge sample identified as *Haliclona* (*Soestella*) sp., showed an irregular growth form with mammiform and volcano-shaped processes. Ocules were abundant and scattered on the surface. The sponge was firm to the touch, and the consistency was compressible and fragile. The colour ranged from greenish black to orange in live specimens but turned light brown in preservative. The coanosomal skeleton had irregular reticulation of primary lines ascending toward the surface, connected by unispicular secondary lines. The ectosomal skeleton consisted of multispicular polygonal meshes. Only oxeas were present that measured 99.5-126 (110.4) × 2.6-5.2 (4.97) *μ*m (Figure 1).

### 3.2. Effects of the HSCE on Nonfunctional and Functional Immunological Parameters

#### 3.2.1. *In Vivo* Immunological Parameters

**Figure 2:**
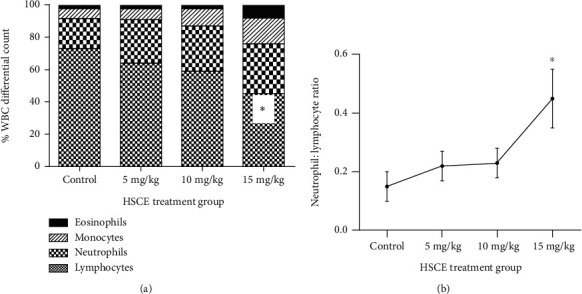
Effect of oral administration of the *Haliclona* (*Soestella*) sp. sponge crude extract (HSCE), once daily for 14 consecutive days in adult male Wistar rats: (a) differential white blood cell counts (WBC/DC); (b) neutrophil : lymphocyte ratio. Results are expressed as mean ± SEM (*n* = 6/group). ^∗^*p* < 0.05 (Mann-Whitney *U* test).

Compared with the control group, the 15 mg/kg HSCE-treated group manifested a significant decrease in total WBC (by 17%; *p* < 0.01), lymphocyte (by 38%), platelet (by 52%), BMC (by 60%) and splenocyte (by 20%) counts (*p* < 0.001), and the splenosomatic index (by 50%; *p* < 0.001) [Table 1; Figure 2(a)], with a concurrent increase in the neutrophil : lymphocyte ratio (*p* < 0.05). This was reiterated in the group treated with the 10 mg/kg dose. The lowest HSCE dose (5 mg/kg) tested showed no effect on these parameters with the exception of a significantly decreased BMC count (*p* < 0.001). Importantly, the BMC count was suppressed by 45%, 45%, and 60% with HSCE doses of 5, 10, and 15 mg/kg (*p* < 0.001), respectively. Furthermore, compared to the control, the neutrophil : lymphocyte ratio showed a significant increase with the 15 mg/kg HSCE treatment [*p* < 0.05; Figure 2(b)]. Though not a parameter associated with the immune status, the RBC count too was significantly depleted (by 53%) with the 15 mg/kg HSCE treatment (*p* < 0.001).

**Figure 3:**
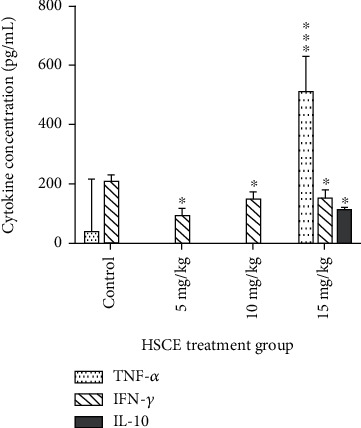
Effect of oral administration of the *Haliclona* (*Soestella*) sp. sponge crude extract (HSCE) once daily for 14 consecutive days on rat plasma cytokine levels of TNF-*α*, IFN-*γ*, and IL-10. Results are expressed as mean ± SEM (*n* = 6/group). ^∗^*p* < 0.05 and ^∗∗∗^*p* < 0.001 (Mann-Whitney *U* test).

Plasma cytokine levels of IFN-*γ*, TNF-*α*, IL-10, and IL-6 of the rats treated with 5, 10, and 15 mg/kg doses of the HSCE were reported at varying degrees, reiterating the immunomodulatory effect of the HSCE. Plasma TNF-*α* level was exclusively reported in the test group treated with the 15 mg/kg HSCE dose, with a significant tenfold increase compared to the control (*p* < 0.001). Importantly, in comparison to the control, IFN-*γ* level significantly decreased with all tested doses (*p* < 0.05; Figure 3). IL-10 was not reported except in the highest HSCE treatment dose (15 mg/kg). The Th_1_ : Th_2_ cytokine ratio, represented by IFN-*γ* : IL-10 (30.61; *p* < 0.05) and TNF-*α* : IL-10 (115.3; *p* < 0.01), calculated for rats treated with the 15 mg/kg dose, was indicative of an unambiguous Th1-skewed cytokine response. Furthermore, compared with the control group, plasma IL-6 level was significantly higher in the 10 mg/mL HSCE treatment (*p* < 0.05), which however was exacerbated twofold in rats treated with the 15 mg/mL HSCE dose (*p* < 0.01).

#### 3.2.2. *Ex Vivo* BMC and Splenocyte Proliferation by the MTT Assay

**Figure 4:**
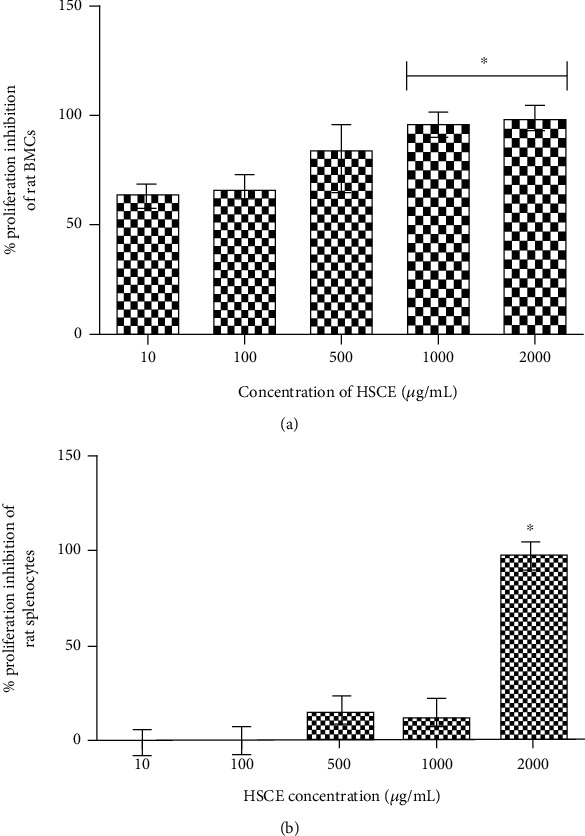
Effect of *Haliclona* (*Soestella*) sp. sponge crude extract (HSCE) on *ex vivo* proliferation of rat (a) bone marrow cells (BMCs) and (b) splenocytes (^∗^*p* < 0.01; Mann-Whitney *U* test).

A dose-dependent percentage inhibition of *ex vivo* rat BMC proliferation by the HSCE was reported in the MTT assay (*p* < 0.05), while a 100% proliferation inhibition was observed in 1000 and 2000 *μ*g/mL HSCE concentrations [*p* < 0.01; Figure 4(a)]. The calculated IC_50_ value was 0.719 *μ*g/mL. A dose-dependent reduction was also observed in the *ex vivo* rat splenocyte proliferation percentage as a measure of the MTT assay compared with the control, with approximately 20% proliferation reduction in 500 and 1000 *μ*g/mL HSCE doses, while this reduction was significant at the 2000 *μ*g/mL concentration (100%) with an IC_50_ value of 0.931 *μ*g/mL [*p* < 0.01; Figure 4(b)].

#### 3.2.3. *Ex Vivo* Cytokine Production by Wistar Rat BMCs

**Figure 5:**
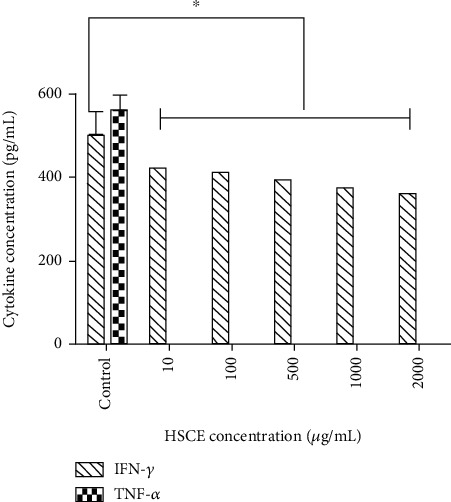
Effect of the *Haliclona* (*Soestella*) sp. sponge crude extract (HSCE) on *ex vivo* cytokine secretion by rat bone marrow cells (BMCs) (^∗^*p* < 0.05; Mann‐Whitney*U*test) (IFN-*γ*: interferon gamma; TNF-*α*: tumour necrosis factor alpha).

The highest IFN-*γ* and TNF-*α* levels were reported by rat BMCs in the controls while all HSCE treatments showed significantly lower IFN*γ* levels (*p* < 0.05; Figure 5). TNF-*α* was completely suppressed by all concentrations of the HSCE-treated BMCs. IL-10 was not reported including in the control group.

### 3.3. Subacute Toxicity of the HSCE

Administration of the highest dose (15 mg/kg) of HSCE once daily for 14 consecutive days did not provoke any overt signs of toxicity in the Wistar rat model; neither hepatotoxic effects with respect to serum ALT, AST, and total protein concentrations, nor nephrotoxic effects with respect to urea and creatinine concentrations were evident as compared with the control (*p* > 0.05; Mann-Whitney *U* test; Table 2). Increased serum cortisol level in rats treated with the highest dose of HSCE (2.08 ± 0.34 ng/mL) vs. the control (1.5 ± 0.21 ng/mL) was recorded, which however was not statistically significant (*p* > 0.05). Compared to the control group, changes in rat body weight were absent with the HSCE treatment (*p* > 0.05).

## 4. Discussion

The global marine pharmaceutical pipeline records several hundred novel marine compounds tested in preclinical trials (phases I, II, and III) annually, which continue to feed the clinical pipeline with potentially valuable compounds; in 2010, this included 4 (3 FDA and 1 EU) approved drugs, 13 in clinical development, 1458 in the preclinical pipeline, and chemical investigation of 8940 marine natural products [23], whereas in 2019, the corresponding numbers were 9, 31, >1500, and >10,000 (https://www.youtube.com/watch?v=vSH_q4ab1hQ&t=10s).

In the current scenario, sponges are considered the most important source of biologically active natural marine products to be investigated for pharmacotherapeutic purposes [6]; several sponge-derived compounds are approved as drugs by the FDA while many others are lined up in the preclinical and clinical trials to be tested against cancer, microbial infections, inflammation, and other diseases [4]. The first compounds isolated from the marine sponge *Tethya crypta*, led to the synthesis of the anticancer drug, Ara-C, and the first and the only sponge-derived compound approved by the FDA as an antiviral drug, Ara-A [24]. The analog of Ara-C is the active ingredient in the drug Cytosar-U®, which is used to treat leukemia and lymphoma [25]. Further, a sesquiterpenoid hydroquinone, isolated from *Dysidea avara*, was developed as a drug for cancer and inflammation [26]. Halichondrin B-3, obtained from sponges such as *Halichondria okadai*, *Axinella* sp. and *Phakellia* sp., is also used to develop anticancer drugs [27–29]. The formulation of eribulin mesylate, which was approved in 2010, is a simplified analog of Halichondrin B [30] and available in the market under the trade name Halaven®, used as a chemotherapeutic for metastatic breast cancer [31].

The search for pharmacological properties of secondary metabolites of marine sponges has resulted in a plethora of therapeutic activity; antibacterial, antiviral, antifungal, antimalarial, anthelminthic, antitumor, anti-inflammatory, immunosuppressive, muscle relaxant, and cardiovascular activity are attributed mainly to the special carbon skeletons of these compounds that interfere with pathogenesis in different sites of the human body [4].

Modulation of the immune system is defined as any change in the immune response that can involve induction, expression, amplification, or inhibition of any phase of the immune response; thus, immunomodulators are substances used for producing effects on the immune system, and based on their effects, are generally of two types: immunosuppressants and immunostimulants [32]. Immunostimulants increase the body's resistance against infection while the latter is a group of heterogenous drugs, which are useful to treat rejection of organ transplants and autoimmune diseases. Marine pharmacognosy has identified a considerable number of immunomodulatory drug leads [33], including those from marine sponges [12, 34–37]. The present study for the first time reports the immunomodulatory activity of a marine sponge of the genus *Haliclona*, *i.e*., of the crude extract of *Haliclona* (*Soestella*) sp. (HSCE) collected from the marine waters of Sri Lanka and tested in the Wistar rat model.

Marine sponges of the family Haliclonidae, to which the genus *Haliclona* belongs to, are reported to contain a rich source of nitrogen-containing metabolites such as halitoxin, xestospongins, sarains, papuamines, and haliclonadiamine, resulting in various biological activities [11]. They are well known for producing a variety of secondary metabolites, particularly, alkaloids [11, 38]. These alkaloids such as manzamine A-D have exhibited prominent antitumour and cytotoxic properties [11, 39]. A wide range of bioactivity such as antimicrobial, hemolytic, hemagglutination, antibacterial, antioxidant, anti-inflammatory, and anticancer activities associated with a variety of alkaloids are also reported from the genus *Haliclona* [11]. Other than alkaloids, the sponges of genus *Haliclona* contain polycaetylinic compounds, sphingosine derivatives, and many miscellaneous compounds, which may result in an array of bioactivity [11]. The taxonomic identification of the sponge species under investigation suggests that it is a novel species, which is reported from Sri Lanka.

The HSCE showed potent dose-dependent immunosuppressive activity with respect to selected nonfunctional and functional immunological tests. The *in vivo* immunosuppressive effects of the highest dose of HSCE (15 mg/kg body weight) tested in the rat model were affirmed by significantly depleted hematological parameters, *i.e*., RBC, total WBC, lymphocyte, and platelet counts, accompanied by diminished cellularity of the bone marrow and of the spleen. Importantly, this dose of the HSCE was devoid of general toxicity, hepatotoxicity, and nephrotoxity in the rat model. A plausible explanation for the immunosuppressive effects thus demonstrated may be attributed to consequences of the 60% suppressed BMC cellularity, evidently suggestive of myelosuppression. Pancytopenia (an abnormal reduction in the number of RBC, WBC, and platelets) can be caused by a wide variety of etiologies, including cytotoxic compounds and drugs [40]. This phenomenon is common in immune dysfunction, especially in immunosuppression. Conversely, thrombocytopenia is often attributed to hypersplenism; the increased pooling of platelets in a spleen enlarged by congestive splenomegaly [41]. In general, approximately one-third of the platelets are sequestered in the spleen and an enlarged spleen results in a low number of platelets circulating in blood [42]. However, the 50% suppressed splenosomatic index (SW/BW) by the highest HSCE dose (15 mg/kg) indicated the absence of splenomegaly, on the contrary atrophy of the spleen, signifying the absence of platelet aggregation in the rat spleen. Thus, the resultant thrombocytopenic condition may be attributed to the low production of platelets by the bone marrow by suppression of hematopoiesis in the BM. The immunosuppressive ramification of the HSCE was further supported by the clear *ex vivo* inhibition of rat BMC and splenocyte proliferation by the HSCE. It would have been prudent to include an established immunosuppressant as a positive control in the experiments, which would have provided better insight into the potential of the *Haliclona* (*Soestella*) sp. as an immunosuppressant marine sponge species.

Thymus and spleen weights are considered to be sensitive indicators of immune stimulation or suppression, stress, and physiologic perturbations. Furthermore, histopathological changes in the spleen and thymus correlate to organ weight changes [43]. Compared to the control group, the significantly reduced spleen to body weight ratio in the 15 mg/kg HSCE-treated rats may have been indicative of the low splenocyte counts.

A significantly elevated neutrophil : lymphocyte ratio (NLR) was reported in Wistar rats treated with the HSCE (15 mg/mL). Increase of NLR is usually observed in animals experiencing oxidative stress which leads to inflammation [44]. The Th1-skewed proinflammatory plasma cytokine response was clearly evident in rats orally gavaged with the high dose of HSCE, as discussed below.

In a normal, healthy immune system, Th_1_ and Th_2_ subsets and related cytokines are systemically well balanced to maintain homeostasis. The *in vivo* and *ex vivo* ability of the HSCE to modulate Th1 and Th2 type cytokines in the rat model was evident. The Th_1_ : Th_2_ cytokine ratio, specifically the TNF-*α* : IL-10 calculated for the *in vivo* treatment of the 15 mg dose of HSCE, with a tenfold increased plasma TNF-*α* level than that of the control, unequivocally indicated a strong Th1-polarized proinflammatory cytokine response. TNF-*α* is listed as a cytokine with myelosuppressive activity [45]. Compared to the controls, the high dose of HSCE also induced a twofold increase in rat plasma IL-6 levels. It has been reported that IL-6 levels increase in patients with chemotherapy-induced myelosuppression [46]. As such, the high rat plasma levels of both TNF-*α* and IL-6 detected may well be associated with the myelosuppressive effect evoked by the 15 mg/kg HSCE dosage. Modulation of cytokine profiles is usually associated with the alteration of the NF-*κ*B pathway [47]. Stimulation of the NF- *κ*B pathway results in the production of proinflammatory cytokines such as TNF-*α* and IFN-*γ*, leading to Th_1_-biased immunomodulation of cytokines as expressed *in vivo* by Wistar rats treated with the HSCE. With experimental evidence established from this study, it may be hypothesised that the activation of the NF-*κ*B pathway triggered by the HSCE that leads to a profuse increase in TNF alpha and IL-6 levels may have induced myelosuppression in the rat model.

Sponge chemosystematics may rely on secondary metabolites as useful biochemical markers. It is plausible that the zoocompounds contained in the HSCE either individually or in synergy mediated the immunosuppression/myelosuppression and the Th1-skewed proinflammatory cytokine responses in the Wistar rat model. Nevertheless, it is imperative to subject the HSCE to activity-guided fractionation, followed by LC-MS and NMR of the active fraction(s) to arrive at a decisive conclusion on structure elucidation of the active compounds. As reported in previous literature, sponges of the genus *Haliclona* contain alkaloids [11]. Furthermore, at least 190 metabolites with antifouling, antimicrobial, antifungal, antimalarial, and cytotoxic activities were isolated from marine sponges of the genus *Haliclona* [48].

In this landscape, the “sponge holobiont,” the association of the diverse, and abundant microorganism consortia as symbionts with marine sponges cannot be disregarded; many sponge-derived natural compounds found to be therapeutically important are suspected to be of such microbial origin [49, 50]. Over 50% of the 52 bacteria species isolated from *Haliclona simulans*, from the west coast of Ireland, exhibited antimicrobial activity [51]. A fungal strain of *Emericella variecolor* isolated from the sponge *Haliclona valliculata* was reported with two novel bioactive natural products, evariquinone and isoemericellin; evariquinone was found to be a strong antiproliferative compound [52]. Therefore, although utmost care was taken to prepare a “clean” HSCE sponge extract, whether the immunomodulatory activity demonstrated by the HSCE is derived from secondary metabolites of the *Haliclona* (*Soestella*) sp., the sponge itself, or whether these are due to derivatives of its associated microbiota, is a matter of conjecture.

The way forward with the current study is clearly two pronged; molecular investigations focussed on DNA barcoding of *Haliclona* (*Soestella*) sp. to establish its nomenclature, and comprehensive sponge chemosystematic studies carried out in parallel, consisting of activity-guided fractionation of the HSCE, followed by structure elucidation of active compound(s) to identify potential immunosuppressant drug leads.

## 5. Conclusions

The present study for the first time reports the immunomodulatory activity of a marine sponge of the genus *Haliclona*. This comprehensive study established that the crude extract of a presumably novel marine sponge species, *Haliclona* (*Soestella*) sp. (HSCE), from Sri Lanka, effectively suppressed both nonfunctional and functional immune responses, such as *in vivo* suppression of the immune cell profile (total WBC, lymphocyte, platelet, splenocyte, and bone marrow cell counts) and the splenosomatic index, and *ex vivo* bone marrow cell and splenocyte proliferation in the Wistar rat model. Collectively, the HSCE was orally active, devoid of general toxic, hepatotoxic, and nephrotoxic effects for a period of 14 days and demonstrated potent immunosuppressant activity (specifically myelosuppression) with a concurrent Th1-skewed cytokine response. Therefore, the HSCE holds much promise as a potential immunosuppressant drug lead.

## Figures and Tables

**Table 1:** Effects of *Haliclona* (*Soestella*) sp. sponge crude extract in the Wistar rat.

Treatment group (*n* = 6/group)	WBC (x10^5^mL^−1^)	Platelets (x10^6^mL^−1^)	Total BMC^#^ (x10^6^mL^−1^)	Splenocytes (x10^6^mL^‐1^)	Splenosomatic index^+^
Control (5% ethanol)	152.7 ± 15.8	387.3 ± 20.8	876.3 ± 17.8	1237.5 ± 46.6	0.002 ± 0.0
HSCE (mg/kg)					
5	132.8 ± 12.3	342 ± 17.2	480 ± 16.4^∗∗∗^	1125 ± 45.3	0.002 ± 0.0
10	130.2 ± 13.1^∗^	289 ± 34.2^∗∗∗^	478 ± 23.4^∗∗∗^	1034.4 ± 35.2^∗∗∗^	0.001 ± 0.0^∗∗∗^
15	125.2 ± 12.2^∗∗^	187 ± 23.5^∗∗∗^	353 ± 12.4^∗∗∗^	994.5 ± 28^∗∗∗^	0.001 ± 0.0^∗∗∗^

^#^BMC—bone marrow cells. ^+^Splenosomaticindex = (spleenweight/bodyweight). HSCE: *Haliclona* (*Soestella*) sp. sponge crude extract. Data presented as mean ± SEM; ^∗^*p* < 0.05, ^∗∗^*p* < 0.01, and ^∗∗∗^*p* < 0.001 (Mann-Whitney *U* test).

**Table 2:** Effect of oral administration of the HSCE on hepatic and renal parameters of rats.

Test parameter	Control (5% ethanol)	HSCE dose : 15 mg/kg
Hepatic parameters		
Alanine aminotransferase (ALT; U/L)	0.54 ± 0.01	0.59 ± 0.00
Aspartate aminotransferase (AST; U/L)	11.56 ± 0.04	12.8 ± 0.03
Total serum proteins (mg/L)	5.70 ± 0.00	5.90 ± 0.02
Bilirubin (U/L)	0.57 ± 0.01	0.9 ± 0.02
Renal parameters		
Urea (mg/mL)	31.5 ± 0.11	32.1 ± 0.01
Creatinine (*μ*mol/L)	46.7 ± 0.00	46.7 ± 0.00

*p* > 0.05.

## Data Availability

The immunological data (cell counts, concentrations of cytokines, etc.) used to support the findings of this study are available from the corresponding author upon request.
